# Strong associations between chromosomal aberrations in blood lymphocytes and the risk of urothelial and squamous cell carcinoma of the bladder

**DOI:** 10.1038/s41598-017-13976-y

**Published:** 2017-10-18

**Authors:** Hongkun Wang, Ying Wang, Krishna K. Kota, Bing Sun, Bhaskar Kallakury, Nabiel N. Mikhail, Douaa Sayed, Ahmed Mokhtar, Doaa Maximous, Etemad H. Yassin, Scarlett X. Sun, Xiaofei Chen, Christopher A. Loffredo, Yun-Ling Zheng

**Affiliations:** 1 Department of Biostatistics, Bioinformatics, and Biomathematics, Lombardi Comprehensive Cancer Center, Georgetown University Medical Center, Washington DC, USA; 2 Cancer Prevention and Control Program, Lombardi Comprehensive Cancer Center, Georgetown University Medical Center, Washington DC, USA; 3 Department of Pathology, Lombardi Comprehensive Cancer Center, Georgetown University Medical Center, Washington DC, USA; 4 South Egypt Cancer Institute, Assiut University, Assiut, Egypt

## Abstract

Chromosomal aberrations (CAs) in blood lymphocytes have been shown to be associated with overall cancer risk and aging. However, their relationship to bladder cancer risk remains to be elucidated. In a case-control study of bladder cancer in Egypt, we examined the relationship between the increased frequency of CAs in blood lymphocytes and bladder cancer risk. High frequency of CAs was significantly associated with an increased risk of bladder cancer [adjusted odds ratios (OR) = 3.90, 95% confidence interval (CI) = 2.65–5.73]. The associations were somewhat stronger in squamous cell carcinomas (SCC, OR = 4.90) than in urothelial carcinomas (UC, OR = 3.62). We also identified chromosome specific CAs for chromosomes 3, 4, 5, 8, 9, 10, 11, 12, 17, 19 that were significantly associated with an increased risk of bladder cancer. We observed particularly strong associations between aberrations of chromosomes 12, 13, 17 and risk of SCC (OR = 7.06, 6.91 and 6.23, respectively). Conclusion: increased frequency of chromosomal aberrations in blood lymphocytes was significantly associated with bladder cancer risk. Overall and chromosome specific aberrations in blood lymphocytes may be a unique set of biomarkers for risk assessments of SCC and UC.

## Introduction

Urinary bladder cancer ranks seventh overall among men worldwide but seventeenth among women^1^. Urinary bladder cancer subtypes include urothelial carcinoma (UC), squamous cell carcinoma (SCC), adenocarcinoma, anaplastic, and other rare forms. The most prevalent subtype is UC, accounting for about 90% of incident cases in developed countries^2^. The genome of bladder cancer cells, like many other human solid tumors, is characterized by chromosomal instability, including structure and numerical chromosomal aberrations (CAs)^3,4^. The formation of structural chromosomal aberration is often associated with carcinogen-induced DNA damage or faulty replication of a damaged DNA template^5^. The capacity to repair damaged-DNA also plays a critical role in the formation of structural CAs^6^. It has long been hypothesized that the level of genetic damages assessed in blood cells reflects the level of damage in the somatic cells affected by carcinogenesis.

The frequency of chromosomal aberrations in peripheral blood lymphocytes has been used for the surveillance of healthy individuals exposed to known carcinogens^7^ and has also been shown to be associated with aging and an increased risk of leukemia^8^. Several epidemiological studies provided consistent evidence that CA frequency in blood lymphocytes was associated with elevated overall cancer risk, independent of other cancer risk factors such as exposure to carcinogens, gender, and age^8–13^. The strongest association was found in melanoma and carcinomas arising from lung, stomach, liver, and prostate^10,11,13^. However, the number of bladder cancer cases in these previous studies was very small (N < 60), thus lacking statistical power to draw meaningful conclusions. In the present report, we examined the association between the frequency of CAs in blood lymphocytes and bladder cancer risk in a large case-control study that included sufficient numbers of both UC and SCC patients to examine the two subtypes of cancer separately (533 cases and 560 controls). The large sample size also allowed us to examine the associations between chromosome specific CAs in blood lymphocytes and bladder cancer risk for the first time.

## Results

### Characteristics of study population

Table 1 summarizes selected demographic characteristics of the study subjects. Comparing with all control subjects, there were no significant differences in the distributions of age, gender, and marital status among all the bladder cancer subjects, while SCC cases were younger (p = 0.0007) and had more female subjects (p = 0.01), while UC cases had more male subjects (p = 0.025). The bladder cancer cases were significantly more likely than the controls to be smokers (73.4% vs 52.5%, p < 0.001). The cases also had lower education levels (p < 0.0001) and lower mean BMI (p < 0.001) compared with controls.

**Table 1:** Demographic characteristics of study subjects.

	All Cases N = 533	Controls N = 560	SCC Cases N = 125	UC Cases N = 394	P	P(SCC)	P(UC)
Age, mean (SD)	59.3(10.9)	60.1(11.5)	56.3(10.3)	60.4(11.0)	0.25	<0.001	0.70
BMI, mean(SD)	24.8(3.8)	26.2(4.8)	24.0(3.7)	25.0(3.9)	<0.001	<0.001	<0.001
**Gender, N (%)**							
Male	428(80.3)	441(78.8)	85(68.0)	333(84.5)			
Female	105(19.7)	119(21.3)	40(32.0)	61(15.5)	0.53	0.010	0.025
**Age Distribution, N (%)**							
≤55	198(37.2)	183(32.7)	59(47.2)	131(33.3)			
56–60	91(17.1)	105(18.8)	21(16.8)	67(17.0)			
61–67	122(22.9)	136(24.3)	28(22.4)	93(23.6)			
>68	122(22.9)	136(24.3)	17(13.6)	103(26.1)	0.48	0.008	0.86
**Residence, N (%)**							
Urban	67(12.6)	29(5.2)	6(4.8)	56(14.2)			
Rural	466(87.4)	530(94.8)	119(95.2)	338(85.8)	<0.001	0.86	<0.001
**Education, N (%)**							
illiteracy	368(69.2)	263(47.2)	100(80.0)	259(65.9)			
Kottab, Literacy class, or Primary school	120(22.6)	151(27.1)	17(13.6)	99(25.2)			
Preparatory or secondary school	41(7.7)	119(21.4)	8(6.4)	32(8.1)			
College or university	3(0.6)	24(4.3)	0(0.0)	3(0.8)	<0.001	<0.001	<0.001
**Marital Status, N (%)**							
Didn’t marry	10(1.9)	9(1.6)	4(3.2)	6(1.5)			
Married	466(87.4)	493(88.0)	105(84.0)	349(88.6)			
Widowed	55(10.3)	56(10.0)	15(12.0)	38(9.6)			
Divorced or separated	2(0.4)	2(0.4)	1(0.8)	1(0.3)	0.98	0.49	0.99
**Tobacco Use, N (%)**							
None User	142(26.6)	266(47.5)	43(34.4)	94(23.9)			
Water pipe only	47(8.8)	52(9.3)	13(10.4)	33(8.4)			
Cigarette only	289(54.2)	192(34.3)	60(48.0)	222(56.4)			
Both	55(10.3)	50(8.9)	9(7.2)	45(11.4)	<0.001	0.022	<0.001
**Schitosomiasis, N (%)**							
No	211(44.7)	280(53.0)	43(38.7)	163(46.7)			
Yes	261(55.3)	248(47.0)	68(61.3)	186(53.3)	0.009	0.006	0.07

SCC = Squamous cell carcinoma; UC = urothelial carcinoma; BMI = body mass index.

### Association between frequency of blood lymphocytes with CAs and bladder cancer risk

The bladder cancer cases were significantly more likely than the controls to have blood lymphocytes with any CAs (59.5% cases vs 44.3% controls, p < 0.0001, Table 2). About a third of cases had 2 or more blood lymphocytes with CAs compared with 11.8% of controls (p < 0.001, Table 2). These patterns of case-control differences in the frequency of blood lymphocytes with CAs remained highly significant for both UC cases and SCC cases. When the study subjects were stratified by age, gender, cigarette smoking status and Schistosoma infection, we observed similar significant patterns of higher frequency of blood lymphocytes with CAs in higher percentage of cases than controls in each sub-category of the study population (Table 2).

**Table 2:** Case-control comparison of frequency of blood lymphocytes with chromosome aberrations, stratified by age, gender, smoking status, and Schistosomiasis status.

No. of lymphocytes with CAs	Cases	Controls	Cases (SCC)	Cases (UC)	P	P (SCC)	P(UC)
**All Subject**	**533**	**560**	**125**	**394**			
0	216(40.5)	312(55.7)	43(34.4)	167(42.4)			
1	150(28.1)	182(32.5)	42(33.6)	106(26.9)			
>=2	167(31.3)	66(11.8)	40(32.0)	121(30.7)	<0.001	<0.001	<0.001
**Male**	**428**	**441**	**85**	**333**			
0	173(40.4)	254(57.6)	31(36.5)	138(41.4)			
1	114(26.6)	136(30.8)	25(29.4)	87(26.1)			
>=2	141(32.9)	51(11.6)	29(34.1)	108(32.4)	<0.001	<0.001	<0.001
**Female**	**105**	**119**	**40**	**61**			
0	43(41.0)	58(48.7)	12(30.0)	29(47.5)			
1	36(34.3)	46(38.7)	17(42.5)	19(31.2)			
>=2	26(24.8)	15(12.6)	11(27.5)	13(21.3)	0.063	0.038	0.27
**Age **<= 60	**289**	**288**	**80**	**198**			
0	109(37.7)	162(56.3)	25(31.3)	81(40.9)			
1	90(31.1)	98(34.0)	31(38.8)	57(28.8)			
>=2	90(31.1)	28(9.7)	24(30.0)	60(30.3)	<0.001	<0.001	<0.001
**Age** >60	**244**	**272**	**45**	**196**			
0	107(43.9)	150(55.2)	18(40.0)	86(43.9)			
1	60(24.6)	84(30.9)	11(24.4)	49(25.0)			
>=2	77(31.6)	38(14.0)	16(35.6)	61(31.1)	<0.001	0.002	<0.001
**Non-tobacco user**	**142**	**266**	**43**	**94**			
0	56(39.4)	143(53.8)	14(32.6)	40(42.6)			
1	48(33.8)	90(33.8)	18(41.9)	29(30.9)			
>=2	38(26.8)	33(12.4)	11(25.6)	25(26.6)	<0.001	0.015	0.005
**Tobacco user**	**391**	**294**	**82**	**300**			
0	160(40.9)	169(57.5)	29(35.4)	127(42.3)			
1	102(26.1)	92(31.3)	24(29.3)	77(25.7)			
>=2	129(33.0)	33(11.2)	29(35.4)	96(32.0)	<0.001	<0.001	<0.001
**Non-Schitosomiasis**	**211**	**280**	**43**	**163**			
0	83(39.3)	154(55.0)	12(27.9)	69(42.3)			
1	69(32.7)	92(32.9)	22(51.2)	46(28.2)			
>=2	59(28.0)	34(12.1)	9(20.9)	48(29.4)	<0.001	0.004	<0.001
**Schitosomiasis**	**261**	**248**	**68**	**186**			
0	111(42.5)	134(54.0)	28(41.2)	80(43.0)			
1	66(25.3)	84(33.9)	15(22.1)	50(26.9)			
>=2	84(32.2)	30(12.1.)	25(36.7)	56(30.1)	<0.001	<0.001	<0.001

CA = Chromosome aberration; SCC = Squamous cell carcinoma; UC = urothelial carcinoma.

Multivariate logistic regression analyses were conducted to further assess the association between frequency of blood lymphocytes with CAs and bladder cancer risk. Higher frequency of blood lymphocytes with CAs was significantly associated with an elevated risk of bladder cancer overall, with an adjusted odds ratio (OR) of 3.90 (95% CI = 2.65–5.73, Table 3). The risk association was slightly higher in younger subjects (age < = 60, OR = 4.68, 95% CI = 2.60–8.42) than in older subjects (OR = 3.36, 95% CI = 2.00–5.61), in males (OR = 4.43, 95% CI = 2.87–6.83) than in females (OR = 3.77, 95% CI = 1.43–9.92), and in smokers (OR = 4.18, 95% CI = 2.46–7.11) than in non-smokers (OR = 3.66, 95% CI = 1.82–7.34). The risk associations between frequency of blood lymphocytes with CAs and bladder cancer risk were stronger in SCC (OR = 4.90, CI = 2.69–8.94) than in UC (OR = 3.62, CI = 2.42–5.41, Table 3).

**Table 3:** Risk association between frequency of blood lymphocytes with CAs and bladder cancer.

	ALL Cases		SCC Cases		UC Cases	
	OR (95% CI)	p-for-trend	OR (95% CI)	p-for-trend	OR (95% CI)	p-for-trend
**All subject**						
0	ref		ref		ref	
1	1.16(0.85–1.57)		1.41(0.84–2.37)		1.06(0.76–1.48)	
>=2	3.90(2.65–5.73)	<0.001	4.90(2.69–8.94)	<0.001	3.62(2.42–5.41)	<0.001
**Age <= 60**						
0	ref		ref		ref	
1	1.20(0.79–1.83)		1.71(0.91–3.23)		0.98(0.62–1.57)	
>=2	4.68(2.60–8.42)	<0.001	4.96(2.23–11.00)	<0.001	4.11(2.21–7.68)	<0.001
**Age** > **60**						
0	ref		ref		ref	
1	1.10(0.70–1.73)		0.88(0.37–2.11)		1.14(0.72–1.84)	
>=2	3.36(2.00–5.61)	<0.001	4.70(1.92–11.49)	0.001	3.26(1.91–5.55)	<0.001
**Male**						
0	ref		ref		ref	
1	1.22(0.86–1.74)		1.30(0.70–2.44)		1.16(0.80–1.68)	
>=2	4.43(2.87–6.83)	<0.001	6.19(3.03–12.63)	<0.001	4.13(2.64–6.47)	<0.001
**Female**						
0	ref		ref		ref	
1	1.06(0.54–2.06)		1.79(0.68–4.71)		0.89(0.41–1.90)	
>=2	3.77(1.43–9.92)	0.021	4.95(1.37–17.93)	0.051	2.88(1.01–8.22)	0.092
**Smokers**						
0	ref		ref		ref	
1	1.20(0.79–1.82)		1.45(0.72–2.92)		1.09(0.70–1.69)	
>=2	4.18(2.46–7.11)	<0.001	5.30(2.40–11.68)	<0.001	4.09(2.37–7.07)	<0.001
**Non-Smokers**						
0	ref		ref		ref	
1	1.33(0.77–2.30)		2.09(0.83–5.27)		1.14(0.62–2.09)	
>=2	3.66(1.82–7.34)	0.001	4.96(1.59–15.46)	0.020	3.25(1.54–6.82)	0.007
**Non-Schitosomiasis**						
0	ref		ref		ref	
1	1.30(0.83,2.02)		2.20(0.96,5.04)		1.09(0.67,1.76)	
>=2	3.65(2.06,6.48)	<0.001	3.74(1.26,11.08)	0.039	3.52(1.95,6.36)	<0.001
**Schitosomiasis**						
0	ref		ref		ref	
1	1.05(0.68,1.64)		1.00(0.47,2.12)		1.05(0.66,1.70)	
>=2	3.38(1.99,5.72)	<0.001	4.52(2.09,9.77)	<0.001	3.12(1.78,5.50)	<0.001

CA = Chromosome aberration; SCC = Squamous cell carcinoma; UC = urothelial carcinoma; CI = confidence interval; ORs were adjusted for age, gender, BMI, smoking status and education; ORs for smokers were adjusted for age, gender, BMI, number of cigarettes smoked and education.

### Association between the frequency of blood lymphocytes with chromosome specific CAs and bladder cancer risk

We examined the association between frequencies of blood lymphocytes with each individual autosomal aberration (autosomes 1–22) and bladder cancer risk. We observed a significantly higher percentage of bladder cancer patients showing CAs in blood lymphocytes for autosomes 3, 4, 5, 8, 9, 10, 11, 12, 17 and 19 (suppl. Table S1). Multivariate logistic regression analyses confirmed that structural aberrations of chromosomes 3, 4, 5, 8, 9, 10, 11, 12, 17 and 19 in blood lymphocytes were significantly associated with bladder cancer risk with ORs ranging from 1.95 to 5.09 (Table 4 and suppl. Table S2). Chromosome 1, 3, 4, 5, 8, 9, 10, 18, 19 aberrations in blood lymphocytes were significantly associated with risk of UC, while chromosome 4, 5, 6, 8, 9, 11, 12, 13, 17, 20 aberrations were significantly associated with risk of SCC (Table 4 and suppl. Table S2). Interestingly, chromosome 12 (OR = 7.06, 95% CI = 2.80–17.76), 13 (OR = 6.91, 95% CI = 2.36–20.23), and 17 (OR = 6.23, 95% CI = 1.54–25.12) aberrations showed strong associations with risk of developing SCC (Table 4 and suppl. Table 2).

**Table 4:** Effect of individual chromosomal aberrations on cancer status.

No. of lymphocytes with CAs	ALL Cases		SCC Cases		UC Cases	
	OR (95% CI)	p	OR (95% CI)	p	OR (95% CI)	p
**Chromosome 3**						
0	Ref		Ref		Ref	
>=1	2.22(1.08–4.54)	0.029	1.87(0.72–4.81)	0.20	2.29(1.07–4.90)	0.033
**Chromosome 4**						
0	Ref		Ref		Ref	
>=1	3.26(1.52–6.98)	0.002	3.32(1.15–9.59)	0.027	3.29(1.49–7.25)	0.003
**Chromosome 5**						
0	Ref		Ref		Ref	
>=1	2.67(1.40–5.11)	0.003	2.30(0.91–5.84)	0.08	2.65(1.35–5.19)	0.005
**Chromosome 8**						
0	Ref		Ref		Ref	
>=1	2.73(1.37–5.46)	0.005	2.88(1.10–5.84)	0.031	2.65(1.28–5.47)	0.009
**Chromosome 9**						
0	Ref		Ref		Ref	
>=1	2.94(1.67–5.15)	<0.001	3.46(1.54–9.58)	0.003	2.77(1.52–5.04)	<0.001
**Chromosome 10**						
0	Ref		Ref		Ref	
>=1	2.97(1.56–5.66)	0.001	2.12(0.72–6.23)	0.17	3.14(1.62–6.08)	<0.001
**Chromosome 11**						
0	Ref		Ref		Ref	
>=1	1.95(0.99–3.86)	0.054	3.85(1.55–9.58)	0.004	1.53(0.74–3.17)	0.26
**Chromosome 12**						
0	Ref		Ref		Ref	
>=1	2.40(1.16–4.96)	0.018	7.06(2.80–17.76)	<0.001	1.69(0.74–3.87)	0.21
**Chromosome 17**						
0	Ref		Ref		Ref	
>=1	2.93(1.04,8.26)	0.043	6.23(1.54,25.12)	0.010	2.20(0.72,6.66)	0.16
**Chromosome 19**						
0	Ref		Ref		Ref	
>=1	5.09(1.07,24.1)	0.040	5.74(0.67,49.15)	0.11	5.09(1.04,25.0)	0.045

CA = Chromosome aberration; SCC = Squamous cell carcinoma; UC = urothelial carcinoma; CI = confidence interval; ORs were adjusted for age, gender, BMI, smoking status and education.

### Association between the frequency of blood lymphocytes with sex chromosome aneuploidy and bladder cancer risk

The percent of females who had >5% lymphocytes with X chromosome aneuploidy was slightly higher in cases (24.8%) than in controls (17.6%, p = 0.19). Chromosome X aneuploidy is extremely rare in males. Multivariate logistic regression analyses revealed that frequency of chromosome X aneuploidy in blood lymphocytes was not significantly associated with bladder cancer risk in females (Table 5). The percent of males who had >5% lymphocytes with Y chromosome aneuploidy was not significantly higher in cases (9.8%) than in controls (8.6%, p = 0.54). The frequency of blood lymphocytes with chromosome Y aneuploidy was not significantly associated with bladder cancer risk in males (Table 6). Structural sex chromosome aberrations were extremely rare in our study population.

**Table 5:** Risk association between frequency of blood lymphocytes with X chromosomal aneuploidy and bladder cancer risk, Female Only.

No. of lymphocytes with X chromosome aneuploidy	Case	Control	OR	(95% CI)	p-for-trend
**All subject**					
0	35	44	ref		
1	44	54	0.96	(0.48–1.93)	
>=2	26	21	1.13	(0.48–2.67)	0.92
**Age <= 60**					
0	27	34	ref		
1	28	38	0.54	(0.22–1.31)	
>=2	18	10	1.41	(0.45–4.39)	0.18
**Age> 60**					
0	8	10	ref		
1	16	16	1.95	(0.48–7.84)	
>=2	8	11	0.65	(0.13–3.42)	0.31
	Case (SCC)	Control	OR	(95% CI)	p-for-trend
**All subject**					
0	9	44	ref		
1	21	54	1.69	(0.59–4.86)	
>=2	10	21	2.05	(0.58–7.25)	0.49
**Age <= 60**					
0	6	34	ref		
1	16	38	1.45	(0.41–5.06)	
>=2	8	10	2.53	(0.53–12.18)	0.51
**Age> 60**					
0	3	10	ref		
1	5	16	2.37	(0.25–22.40)	
>=2	2	11	0.82	(0.04–15.96)	0.61
	Case (UC)	Control	OR	(95% CI)	p-for-trend
**All subject**					
0	25	44	ref		
1	22	54	0.66	(0.30–1.45)	
>=2	14	21	0.76	(0.28–2.05)	0.58
**Age <= 60**					
0	20	34	ref		
1	12	38	0.35	(0.12–0.96)	
>=2	8	10	1.04	(0.29–3.77)	0.09
**Age >60**					
0	5	10	ref		
1	10	16	1.51	(0.32–7.03)	
>=2	6	11	0.50	(0.07–3.33)	0.43

SCC = Squamous cell carcinoma; UC = urothelial carcinoma; CI = confidence interval; ORs were adjusted for age, gender, BMI, smoking status and education.

**Table 6:** Risk association between frequency of blood lymphocytes with Y chromosome aneuploidy and bladder cancer risk, Male Only.

No of lymphocyte with Y chromosome aneuploidy	Case	Control	OR	(95% CI)	p-for-trend
**All subject**					
0	264	307	ref		
1	122	96	1.47	(1.02–2.12)	
>=2	42	38	1.31	(0.74–2.31)	0.10
**Age <= 60**					
0	148	155	ref		
1	58	40	1.46	(0.83–2.56)	
>=2	10	11	0.58	(0.18–1.84)	0.23
**Age >60**					
0	116	152	ref		
1	64	56	0.10	(0.93–2.47)	
>=2	32	27	0.22	(0.89–3.32)	0.11
SCC cases only	Case(SCC)	Control	OR	(95% CI)	p-for-trend
0	49	307	ref		
1	28	96	2.56	(1.36–4.82)	
>= 2	8	38	1.35	(0.49–3.71)	0.01
**Age <= 60**					
0	30	155	ref		
1	18	40	2.99	(1.29–6.97)	
>=2	2	11	0.63	(0.09–4.26)	0.02
**Age >60**					
0	19	152	ref		
1	10	56	2.93	(1.05–8.21)	
>=2	6	27	2.08	(0.57–7.57)	0.10
UC cases only	Case(UC)	Control	OR	(95% CI)	p-for-trend
0	208	307	ref		
1	92	96	1.31	(0.89–1.92)	
>=2	33	38	1.25	(0.69–2.26)	0.36
**Age <= 60**					
0	112	155	ref		
1	39	40	1.21	(0.66–2.25)	
>=2	7	11	0.54	(0.15–1.95)	0.49
**Age >60**					
0	96	152	ref		
1	53	56	1.40	(0.85–2.33)	
>=2	26	27	1.65	(0.84–3.25)	0.21

SCC = Squamous cell carcinoma; UC = urothelial carcinoma; CI = confidence interval; ORs were adjusted for age, gender, BMI, smoking status and education.

### Association between frequency of blood lymphocytes with CAs and selected host factors

There are no significant differences between the frequency of blood lymphocytes with CAs and tumor grade, muscle invasive status and years of smoked cigarettes (suppl. Table S3). The frequency of blood lymphocytes with CAs was significantly higher in smokers who smoked >10 cigarettes per day than in smokers who smoked <10 cigarettes per day (p = 0.019, suppl. Table S3). The frequency of blood lymphocytes with CAs was borderline significantly higher in smokers than in non-smokers (p = 0.074, suppl. Table S3).

## Discussion

In the present study, we showed that a high frequency of blood lymphocytes with structural CAs is significantly associated with an increased risk of bladder cancer and that the risk association is slightly stronger in SCC than in UC. Our results are consistent with findings from previous cohort studies which showed that CA frequency in blood lymphocytes was significantly associated with risk of multiple cancers of other organ sites^8–10,13–16^.

Chromosome aberrations are considered to derive from unrepaired or misrepaired DNA lesions induced by exogenous or endogenous exposures to DNA-damaging agents. An increase in chromosomal aberrations could also be due to genetic or acquired conditions conferring a higher susceptibility to genetic damage^17^. Elevated levels of chromosomal aberrations in blood lymphocytes may be seen as an indicator of the combined effects of environmental exposures and genetic susceptibility to DNA-damage. Thus the occurrence of chromosomal aberrations in patients at the time of diagnosis may reflect the history of relevant events in carcinogenesis, and serve as biomarker for cancer risk. The results of the present study support the hypothesis that chromosomal aberrations in blood lymphocytes are associated with bladder cancer risk.

We evaluated several known bladder cancer risk factors, such as age, gender, tobacco use and schistosomiasis, as potential confounders or effect modifiers. Stratified analyses suggested a stronger risk association between CA in blood lymphocytes and bladder cancer in the younger age group than in the older age group, in smokers than in non-smokers, and in males than in females. However, we found no significant interactions between these factors and CA in blood lymphocytes on bladder cancer risk, suggesting CA in blood lymphocytes is an independent risk factor for bladder cancer. These results are consistent with available literature that points toward that CA in blood lymphocytes is an independent risk factor for cancers at various sites in the body^9–13^.

One of the exceptional strengths of the present study is that chromosomal aberrations were analyzed by karyotyping of G-banded chromosomes. This approach allows the characterization of CA for each of the 23 pairs of human chromosomes. Large sample size provided sufficient power to evaluate the association between chromosome specific CAs and bladder cancer risk. We found that structural CAs of 11 autosomes, namely chromosomes 3, 4, 5, 6, 8, 9, 10, 11, 12, 17, 19, were significantly associated with bladder cancer risk. Three (chromosome 4, 8, 9) of the 11 chromosomes were significantly associated with risk of both UC and SCC. Like many other solid tumors, structure chromosome aberrations are common in bladder tumor cells^18,19^. Previous cytogenetic analyses have shown that loss of chromosomes 1p, 5q, 8p, 9p, 10q, 11p, and 17p and gain of chromosomes 1q, 3q, 5p, 8q, 13q and 17q are common events in bladder tumors^18,19^. We analyzed 424 tumors collected from UC cases by array comparative genome hybridization (CGH) and found that loss of chromosomes 8p, 9p and 17p, and gain of chromosomes 1q, 3p, 5p, 6p, 8q, 10p, 11q, 12q, 14q, 17q and 20q were frequently observed (>25% tumors, data not shown). The present study, for the first time, provided evidence that instability in chromosomes 3, 4, 5, 6, 8, 9, 10, 11, 12, 17, 19 are shared between blood lymphocytes and bladder tumors, suggesting that inherent instabilities in these chromosomes may be a contributing factor to bladder carcinogenesis.

Most interestingly, we found that aberrations in chromosomes 12, 13, and 17 in blood lymphocytes is particularly strong risk factors for SCC. SCC of bladder is rare in the developed world, but is frequent in regions with high prevalence of Schistosoma infection (SH). SH is a well-established risk factor for SCC^20–23^, and is classified as a Group 1 carcinogen^24^. This parasitic disease, characterized by repetitive infections, causes damage to the bladder and kidneys^23,25^. It is thought that bladder tumors occur as a result of chronic inflammation, leading to metaplasia. Previous cytogenetic studies have not found a consistently distinct pattern of chromosome alterations specific for SCC^26–29^. Loss of chromosome 17p has been reported in both UC and SCC tumors, and was found more frequently in SCC tumors^26,29^. There is one small study that reported frequent loss of chromosome 13q in SCC tumors^28^. Frequent alteration of chromosome 12 has not been reported in SCC. Our study is the first to report that chromosome 12, 13, and 17 aberrations in blood lymphocytes are strongly associated with the risk of SCC. These strong associations warrant further investigation.

Sex chromosome aneuploidy, particularly loss of chromosome X or Y, in blood lymphocytes has been shown to be associated with aging^12,30,31^. Loss of chromosome Y in blood lymphocytes has been reported to be associated with non-hematological cancer mortality^12^ and colorectal and prostate cancer risk^32^. However, a recent large cohort study found that mosaic loss of chromosome Y in blood lymphocytes was not consistently associated with overall or specific cancer risk (for example, bladder, lung or prostate cancer) nor with cancer survival after diagnosis^33^. Loss of chromosome Y is a frequent occurrence in bladder tumors^34,35^. In the present study, we found that mosaic loss of chromosome Y in blood lymphocytes was not associated with bladder cancer risks in males. We observed no significant association between loss of chromosome X and bladder cancer risk among females.

A number of strengths are presented in this study. This is one of the largest cytogenetic evaluations undertaken for bladder cancer, which allowed us to assess chromosome specific CAs and identified a set of chromosome specific CAs for UC and SCC separately. The unique epidemiological study setting allowed for evaluation of several known bladder cancer risk factors, such as tobacco smoking and schistosomiasis.

Among the limitations of the current study is the case-control study design. Retrospective case-control study design can not rule out the possibility of reverse causality. However, the blood samples in cases were collected before any treatment was implemented, thus minimizing the effect of treatment on the quality of the blood. The frequency of CAs in blood lymphocytes was not associated with tumor grade and muscle invasiveness (suppl. Table S3), suggesting disease status is not likely the explanation for the observed high frequency of CAs in blood lymphocytes. An additional limitation is that only a moderate number of blood lymphocytes (N = 30) were analyzed: karyotyping is a labor-intensive and time consuming process, and so some low frequency CAs (<5%) may have been missed, leading to under-estimating the level of CAs in blood lymphocytes. Unavailability of dietary data, and thus the potential effect of dietary factors on the frequency of CAs in blood lymphocytes cannot be evaluated.

In conclusion, the present study demonstrated that increased frequency of chromosomal aberrations in blood lymphocytes was significantly associated with bladder cancer risk. This study is the first to reveal that chromosome specific CAs for autosomes 3, 4, 5, 6, 8, 9, 10, 11, 12, 17, 19 in blood lymphocytes were significantly associated with bladder cancer risk. Most interestingly, we observed a particularly strong association between chromosome 12, 13 and 17 aberrations and risk of SCC. If confirmed by future studies, chromosomal aberrations in blood lymphocytes, when used in combination with other risk factors, may become a set of useful biomarkers for bladder cancer risk assessment.

## Materials and Methods

### Study Population

The study population accrual and eligibility criteria were described previously^36^. In brief, bladder cancer cases were recruited from the 2 referral cancer centers in Egypt: the National Cancer Institute in Cairo and the South Egypt Cancer Institute in Assiut. These institutions are the sole tertiary care centers for bladder cancer in their regions. Eligible cases were adults between age 19 and 80 years, self-identified as able to participate in an interview, and diagnosed within 12 months with presumed bladder cancer. Patients who had a prior history of other cancers were excluded. For each case, the pathology report and available H&E slides prepared from the surgical or biopsy specimen of urinary bladder tissue were reviewed by either one of the two study pathologists (B.K. and I.G.) who reported as either (i) urothelial carcinoma (UC), (ii) squamous cell carcinoma (SCC), (iii) adenocarcinoma, or other, including undifferentiated carcinoma. Carcinomas that metastasized to the bladder were excluded. This report includes subjects enrolled between January, 2010 and June, 2014.

Non-cancer controls were randomly selected from the general population to frequency-match the cumulative group of cases on gender and age (5-year interval). All controls fulfilled the following eligibility criteria: (i) no known diagnosis of any cancer; (ii) between ages 19 and 80; and (iii) self-identified as able to participate in an interview. Using a portable ultrasound machine, the physician accompanying the recruitment team conducted an abdominal ultrasound examination to rule out asymptomatic abdominal mass. The interview and phlebotomy were conducted at the participants’ home. The participation rates were 84% for cases and 97% for controls.

After explaining the study and obtaining the consent, trained interviewers administered a structured questionnaire, assessing socio-demographic characteristics including current residence and birth governorate, prior medical history with emphasis on schistosomiasis or other urinary tract infection, cigarette and water pipe smoking status and history, and reproductive history (for women). Histories of exposure to environmental tobacco smoke (ETS) at home and outside the home were also recorded.

The study was approved by the Institutional Review Boards of Georgetown University, the 2 collaborating cancer centers in Egypt, and the National Scientific and Research Ethical Committee at the Egyptian Ministry of Health and Population. All participants signed an informed consent form and donated a blood sample. Socioeconomic characteristics and epidemiological and clinical data were collected through a structured, in-person interview and review of medical records. All experiments were performed in accordance with relevant guidelines and regulations.

### Chromosome preparation from short-term culture of blood lymphocyte

Blood was obtained by trained interviewers in heparinized tubes and blood samples from cases were collected before any treatments were given to the patients. Blood lymphocyte cultures were set up within 48 hours after blood draw, following the protocol as previously described^37^. Briefly, one ml of fresh whole blood was added to 9 ml of RPMI-1640 medium, supplemented with 15% fetal bovine serum, 1.5% of phytohemagglutinin and 100 unites/ml each of penicillin and streptomycin. The blood lymphocytes were cultured at 37 °C for 4 days (92–96 hours) and on the day of harvesting, colcemid (0.2 μg/ml) was added to the culture and incubated at 37 °C for additional one hour. The cells were then treated in a hypotonic solution (0.06 M KCl) and fixed in the fixative (3 parts of methanol with 1 part of acetic acid). The fixed cells were kept at −20 °C for future assays.

### Cytogenetic analysis of chromosomal aberrations

Chromosome preparations were dropped onto clean microscopic slides to make metaphase spreads. The slides were counterstained with 300 ng/ml 4′-6-diamidino-2-phenylindole (DAPI) in an anti-fade mounting medium. The slides were analyzed using an epifluorescence microscope equipped with a charge-coupled device camera. Metaphase cells were captured with exposure times of 0.05 seconds using a DAPI filter under 1000 x magnification. Digitized metaphase images were analyzed using the Isis software (MetaSystems Inc. Boston, MA) with karyotyping capability by DAPI banding (equivalent to G-banding). For each study subject, 30 metaphase cells were randomly selected from one or two (if the number of good metaphase cells on one slide were less than 30) slides and karyotyped. Structural CAs were classified according to the International System for Human Cytogenetic Nomenclature, including chromosomal translocations, deletions, insertions, dicentric chromosomes, ring chromosomes, marker chromosomes, and structurally abnormal chromosomes. The number of metaphase cells showing any CA or any chromosome specific CA was recorded for data analysis. X or Y chromosome aneuploidy was recorded as number of blood lymphocytes with loss or gain of sex chromosomes. Laboratory personnel who were responsible for blood culture and karyotyping were blind to the case-control status of the subjects. One cytogenetist (Y.L.Z) performed karyotyping for the whole project. Repeated analysis of computerized images from ~5% of the subjects showed 100% agreement in scoring the CAs.

### Variable definition and statistical analysis

The primary exposure of interest was the frequency of blood lymphocytes with structural CAs or with sex chromosome aneuploidy. Tobacco use was categorized as “never users”, “waterpipe only”, “cigarette only”, and “both waterpipe and cigarette”. Participants who had smoked less than 100 cigarettes in their lifetime and had never smoked a waterpipe were classified as “never users”; those who smoked less than 100 cigarettes in their lifetime but reported smoking waterpipe were classified as “waterpipe only” users; “cigarette only” users were those who had never smoked waterpipe but had smoked at least 100 cigarettes in their lifetime; and “both waterpipe and cigarette” users were those reported smoking at least 100 cigarettes in their lifetime and also used waterpipe. Schistosoma infections (any during the lifetime) were self-reported.

Student’s t-test and Chi-square test were respectively used to compare continuous variables and categorical variables between cases and controls. We used unconditional logistic regression to assess the risk of bladder cancer with the increased frequency of blood lymphocytes showing CAs. The analyses were stratified by age due to the fact that age has been identified in other studies as an important risk factor associated with cancer risk. All models were adjusted for the matching factors – age and gender. In addition, multivariate models were adjusted for body mass index (BMI), education (none, literacy classes/primary school, preparatory/high/technical school, or college/university), and tobacco use (non-user, waterpipe only, cigarette only, both waterpipe and cigarette). The covariates included in the model were selected based on the matching variables and the bivariate relationship between the covariates and the outcome variable. The penalized likelihood estimates for the odds ratios in the logistic models were reported when data were sparse (where the count in sub-category was 5 or less). *P*-values were two-sided and considered statistically significant if *P* < 0.05. All analyses were performed using SAS software, version 9.3 (SAS Institute, Cary, NC).

## Electronic supplementary material

Supplementary Information
