# Author Correction: Strong associations between chromosomal aberrations in blood lymphocytes and the risk of urothelial and squamous cell carcinoma of the bladder

**DOI:** 10.1038/s41598-018-26488-0

**Published:** 2018-05-22

**Authors:** Hongkun Wang, Ying Wang, Krishna K. Kota, Bing Sun, Bhaskar Kallakury, Nabiel N. Mikhail, Douaa Sayed, Ahmed Mokhtar, Doaa Maximous, Etemad H. Yassin, Scarlett X. Sun, Xiaofei Chen, Christopher A. Loffredo, Yun-Ling Zheng

**Affiliations:** 1 Department of Biostatistics, Bioinformatics, and Biomathematics, Lombardi Comprehensive Cancer Center, Georgetown University Medical Center, Washington DC, USA; 2 Cancer Prevention and Control Program, Lombardi Comprehensive Cancer Center, Georgetown University Medical Center, Washington DC, USA; 3 Department of Pathology, Lombardi Comprehensive Cancer Center, Georgetown University Medical Center, Washington DC, USA; 4 South Egypt Cancer Institute, Assiut University, Assiut, Egypt

Correction to: *Scientific Reports* 10.1038/s41598-017-13976-y, published online 18 October 2017

This Article contains errors. The footnotes of Table 3, Table 5 and Table 6 state that odds ratios in those tables were adjusted for gender. Table 3 has sub-categorical analysis on male or female only, and tables 5 and 6 show results for female only and male only, respectively, so gender was not included in the model adjustment for these results.

The footnote of Table 3 that reads:

“CA = Chromosome aberration; SCC = Squamous cell carcinoma; UC = urothelial carcinoma; CI = confidence interval; ORs were adjusted for age, gender, BMI, smoking status and education; ORs for smokers were adjusted for age, gender, BMI, number of cigarettes smoked and education”.

should read:

“CA = Chromosome aberration; SCC = Squamous cell carcinoma; UC = urothelial carcinoma; CI = confidence interval; ORs were adjusted for age, gender, BMI, smoking status and education; ORs for smokers were adjusted for age, gender (whenever relevant), BMI, number of cigarettes smoked and education”.

The footnote of Table 5 that reads:

“SCC = Squamous cell carcinoma; UC = urothelial carcinoma; CI = confidence interval; ORs were adjusted for age, gender, BMI, smoking status and education”.

should read:

“SCC = Squamous cell carcinoma; UC = urothelial carcinoma; CI = confidence interval; ORs were adjusted for age, BMI, smoking status and education”.

The footnote of Table 6 that reads:

“SCC = Squamous cell carcinoma; UC = urothelial carcinoma; CI = confidence interval; ORs were adjusted for age, gender, BMI, smoking status and education”.

should read:

“SCC = Squamous cell carcinoma; UC = urothelial carcinoma; CI = confidence interval; ORs were adjusted for age, BMI, smoking status and education”.

Additionally, this Article contains an error in the Materials and Methods section, under the subheading ‘Cytogenetic analysis of chromosomal aberrations’.

“X or Y chromosome aneuploidy was recorded as number of blood lymphocytes with loss or gain of sex chromosomes. Laboratory personnel who were responsible for blood culture and karyotyping were blind to the case-control status of the subjects”.

should read:

“X or Y chromosome aneuploidy was recorded as number of blood lymphocytes with loss or gain of sex chromosomes. Chromosome aneuploidy is defined as loss or gain of a whole chromosome in > = 5% of the cells. Laboratory personnel who were responsible for blood culture and karyotyping were blind to the case-control status of the subjects”.

These changes do not affect the conclusions of the article. The authors apologize for the errors.

