# Supplementary material for: Strong associations between chromosomal aberrations in blood lymphocytes and the risk of urothelial and squamous cell carcinoma of the bladder
Source: Sci Rep. 2017 Oct 18;7:13493. doi: 10.1038/s41598-017-13976-y (PMC5647374; doi:10.1038/s41598-017-13976-y)
Supplement: Supplementary file 1 — Supplementary Information [file 41598_2017_13976_MOESM1_ESM.pdf]

Strong associations between chromosomal aberrations in blood lymphocytes and the risk of urothelial and squamous cell carcinoma of the bladder

Hongkun Wang[1],Ying Wang[2], Krishna K. Kota[2], Bing Sun[2], Bhaskar Kallakury[3], Nabiel N. Mikhail[4], Douaa Sayed[4], Ahmed Mokhtar[4], Doaa Maximous[4], Etemad H Yassin[4], Scarlett X. Sun[2], Xiaofei Chen[1] , Christopher A. Loffredo[1,2] and Yun-Ling Zheng[1,2]*

Supplemental Tables

Table S1: Case-control comparison of frequency of blood lymphocytes with CAs, stratified by chromosome

No. of lymphocytes with CAs	Cases	Controls	Cases (SCC)	Cases (UC)	P	? (SCC)	P(UC)
**Chromosome 1**							
0	485(91.5)	529(94.5)	116(93.5)	355(90.6)			
>=1	45(8.5)	31(5.5)	8(6.5)	37(9.4)	0.06	0.69	0.021
**Chromosome 2**							
0	498(93.4)	534(95.4)	118(94.4)	367(93.1)			
>=1	35(6.6)	26(4.6)	7(5.6)	27(6.9)	0.17	0.65	0.14
**Chromosome 3**							
0	504(94.6)	547(97.7)	115(92.0)	375(95.2)			
>=1	29(5.4)	13(2.3)	10(8.0)	19(4.8)	0.007	0.001	0.035
**Chromosome 4**							
0	500(93.8)	547(97.7)	116(92.8)	370(93.9)			
>=1	33(6.2)	13(2.3)	9(7.2)	24(6.1)	0.001	0.005	0.003
**Chromosome 5**							
0	490(91.9)	541(96.6)	115(92.0)	362(91.9)			
>=1	43(8.1)	19(3.4)	10(8.0)	32(8.1)	<0.00	0.021	0.001
**Chromosome 6**							
0	496(93.1)	536(95.7)	114(91.2)	368(93.4)			
>=1	37(6.9)	24(4.3)	11(8.8)	26(6.6)	0.06	0.038	0.11
**Chromosome 7**							
0	490(91.9)	523(93.4)	118(94.4)	359(91.1)			
>=1	43(8.1)	37(6.6)	7(5.6)	35(8.9)	0.35	0.68	0.19
**Chromosome 8**							
0	496(93.1)	543(97.0)	115(92.0)	367(93.2)			
>=1	37(6.9)	17(3.0)	10(8.0)	27(6.8)	0.003	0.010	0.006
**Chromosome 9**							
0	472(88.6)	535(95.5)	108(86.4)	353(89.6)			
>=1	61(11.4)	25(4.5)	17(13.6)	41(10.4)	<0.00	<0.00	<0.001
**Chromosome**							
0	493(92.5)	543(97.0)	118(94.4)	362(91.9)			
>=1	40(7.5)	17(3.0)	7(5.6)	32(8.1)	<0.00	0.16	<0.001
**Chromosome**							
0	500(93.8)	542(96.8)	113(90.4)	373(94.7)			
>=1	33(6.2)	18(3.2)	12(9.6)	21(5.3)	0.020	0.002	0.10
**Chromosome**							
0	502(94.2)	546(97.5)	109(87.2)	379(96.2)			
>=1	31(5.8)	14(2.5)	16(12.8)	15(3.8)	0.006	<.001	0.25
**Chromosome**							
0	510(95.7)	549(98.0)	115(92.0)	381(96.7)			
>=1	23(4.3)	11(2.0)	10(8.0)	13(3.3)	0.025	<0.00	0.19
**Chromosome**							
0	498(93.4)	535(95.5)	116(92.8)	369(93.6)			
>=1	35(6.6)	25(4.5)	9(7.2)	25(6.4)	0.13	0.20	0.20
**Chromosome**							
0	517(97.0)	543(97.0)	120(96.0)	383(97.2)			
>=1	16(3.0)	17(3.0)	5(4.0)	11(2.8)	0.97	0.58	0.83
**Chromosome**							
0	517(97.0)	551(98.4)	122(97.6)	381(96.7)			
>=1	16(3.0)	9(1.6)	3(2.4)	13(3.3)	0.12	0.54	0.09

**Chromosome**								
	0	516((96.8)	553(98.8)	119(95.2)	383(97.2)			
	>=1	17(3.2)	7(1.2)	6(4.8)	11(2.8)	0.029	0.008	0.08
**Chromosome**								
	0	516(96.8)	551(98.4)	125(100)	377(95.7)			
	>=1	17(3.2)	9(1.6)	0(0.0)	17(4.3)	0.09	0.376	0.011
**Chromosome**								
	0	522(97.9)	556(99.3)	122(97.6)	386(98.0)			
	>=1	11(2.1)	4(0.7)	3(2.4)	8(2.0)	0.055	0.090	0.07
**Chromosome**								
	0	515(96.6)	552(98.6)	117(93.6)	384(97.5)			
	>=1	18(3.4)	8(1.4)	8(6.4)	10(2.5)	0.035	0.000	0.21
**Chromosome**								
	0	524(98..3)	555(99.1)	122(97.6)	388(98.5)			
	>=1	9(1.7)	5(0.9)	3(2.4)	6(1.5)	0.24	0.156	0.37
**Chromosome**								
	0	525(98.5)	553(98.8)	121(96.8)	391(99.2)			
	>=1	8(1.5)	7(1.2)	4(3.2)	3(0.8)	0.72	0.116	0.47

CAs = chromosome aberrations; SCC = Squamous cell carcinoma; UC = urothelial carcinoma.

Table S2: Effect of individual chromosomal aberrations on Cancer Status

No. of lymphocytes with CAs		ALL Cases		SCC Cases		UC Cases	
		OR (95% CI)	p-value	OR (95% CI)	p-value	OR (95% CI)	p-value
**Chromosome 1**							
	0	Ref		Ref		Ref	
	>=1	1.64(0.96,2.81)	0.07	1.29(0.53,3.12)	0.57	1.80(1.03,3.13)	0.039
**Chromosome 2**							
	0	Ref		Ref		Ref	
	>=1	1.53(0.83,2.8)	0.17	1.69(0.64,4.47)	0.29	1.32(0.70,2.50)	0.39
**Chromosome 3**							
	0	Ref		Ref		Ref	
	>=1	2.22(1.08,4.54)	0.029	1.87(0.72,4.81)	0.20	2.29(1.07,4.90)	0.033
**Chromosome 4**							
	0	Ref		Ref		Ref	
	>=1	3.26(1.52,6.98)	0.002	3.32(1.15,9.59)	0.027	3.29(1.49,7.25)	0.003
**Chromosome 5**							
	0	Ref		Ref		Ref	
	>=1	2.67(1.40,5.11)	0.003	2.30(0.91,5.84)	0.079	2.65(1.35,5.19)	0.005
**Chromosome 6**							
	0	Ref		Ref		Ref	
	>=1	1.89(1.01,3.57)	0.05	3.36(1.35,8.34)	0.009	1.67(0.85,3.27)	0.13
**Chromosome 7**							
	0	Ref		Ref		Ref	
	>=1	1.15(0.69,1.93)	0.59	0.88(0.36,2.18)	0.78	1.24(0.73,2.12)	0.43
**Chromosome 8**							
	0	Ref		Ref		Ref	
	>=1	2.73(1.37,5.46)	0.004	2.88(1.10,7.53)	0.031	2.65(1.28,5.47)	0.008
**Chromosome 9**							
	0	Ref		Ref		Ref	
	>=1	2.94(1.67,5.15)	<0.001	3.46(1.54,7.74)	0.003	2.77(1.52,5.04)	<0.001
**Chromosome 10**							
	0	Ref		Ref		Ref	
	>=1	2.97(1.56,5.66)	0.001	2.12(0.72,6.23)	0.17	3.14(1.62,6.08)	<0.001
**Chromosome 11**							
	0	Ref		Ref		Ref	
	>=1	1.95(0.99,3.86)	0.05	3.85(1.55,9.58)	0.004	1.53(0.74,3.17)	0.26
**Chromosome 12**							
	0	Ref		Ref		Ref	
	>=1	2.40(1.16,4.96)	0.018	7.06(2.80,17.8)	<.001	1.69(0.74,3.87)	0.21
**Chromosome 13**							
	0	Ref		Ref		Ref	
	>=1	2.09(0.93,4.71)	0.08	6.91(2.36,20.2)	<0.001	1.51(0.60,3.79)	0.38
**Chromosome 14**							
	0	Ref		Ref		Ref	
	>=1	1.70(0.92,3.16)	0.09	2.19(0.88,5.45)	0.091	1.51(0.78,2.94)	0.22
**Chromosome 15**							
	0	Ref		Ref		Ref	
	>=1	1.05(0.5,2.22)	0.89	1.46(0.46,4.65)	0.52	0.94(0.42,2.13)	0.89
**Chromosome 16**							
	0	Ref		Ref		Ref	
	>=1	1.67(0.68,4.07)	0.26	1.40(0.31,6.32)	0.66	1.78(0.71,4.49)	0.22
**Chromosome 17**							
	0	Ref		Ref		Ref	

	>=1	2.93(1.04,8.26)	0.043	6.23(1.54,25.1)	0.010	2.20(0.72,6.66)	0.16
**Chromosome 18**							
	0	Ref		Ref		Ref	
	>=1	2.20(0.85,5.69)	0.10	0.00(0.00,1.00)	0.99	2.76(1.1,6.96)	0.031
**Chromosome 19**							
	0	Ref		Ref		Ref	
	>=1	5.09(1.07,24.1)	0.040	5.74(0.67,49.1)	0.11	5.09(1.04,25.01)	0.045
**Chromosome 20**							
	0	Ref		Ref		Ref	
	>=1	2.21(0.91,5.38)	0.08	6.92(2.18,21.9)	0.001	1.76(0.66,4.74)	0.26
**Chromosome 21**							
	0	Ref		Ref		Ref	
	>=1	1.64(0.50,5.44)	0.42	2.49(0.51,12.0)	0.26	1.26(0.34,4.61)	0.73
**Chromosome 22**							
	0	Ref		Ref		Ref	
	>=1	0.93(0.30,2.84)	0.89	2.40(0.56,10.2)	0.24	0.52(0.12,2.16)	0.37

CAs = chromosome aberrations; SCC = Squamous cell carcinoma; UC = urothelial carcinoma; ORs were adjusted for age, gender, BMI, smoking status and education

Table S3: Association of chromosome aberrations with selected host factors

No. of lymphocytes with CAs	0	1	≥2	P
**Tumor Grade**	**213**	**146**	**166**	
Low	119 (55.9)	82 (56.2)	94 (56.6)	
High	94 (44.1)	64 (43.8)	72 (43.4)	0.99
**Muscle Invasive**	**164**	**119**	**129**	
Yes	38 (23.2)	36 (30.3)	33 (25.6)	
No	126 (76.8)	83 (69.8)	96 (74.4)	0.40
**Smoking Status**	**528**	**332**	**233**	
Yes	281 (53.2)	166 (50.0)	139 (59.7)	
No	247 (46.8)	166 (50.0)	94 (40.3)	0.07
**Years of Cigarette smoking**	**273**	**164**	**133**	
≤35 years	124 (45.4)	83 (50.6)	67 (50.4)	
> 35 years	149 (54.6)	81 (49.4)	66 (49.6)	0.48
**# of Cigarettes smoked/day**	**281**	**165**	**138**	
1-10	98 (34.9)	39 (23.6)	36 (26.1)	
11-20	162 (57.7)	108 (65.5)	96 (69.6)	
>20	21 (7.5)	18 (10.9)	6 (4.4)	0.019
